# Ecological risk assessment of heavy metal contamination and macrobenthos response in the Apies River, South Africa

**DOI:** 10.1007/s10661-026-15348-4

**Published:** 2026-05-04

**Authors:** Jeffrey Lebepe, Moleseng Claude Moshobane, Mapurunyane Callies Selala

**Affiliations:** 1 Department of Biology and Environmental Sciences, Sefako Makgatho Health Sciences University, Pretoria, 0204 South Africa; 2 School of Agriculture and Sciences, University of KwaZulu-Natal, Westville, Durban, 4000 KwaZulu-Natal South Africa; 3 South African National Biodiversity Institute, Pretoria National Botanical Garden, Brummeria, Silverton, 0184 South Africa

**Keywords:** Sediments, Macroinvertebrates, Wastewater effluents, Metal pollution, River in Africa

## Abstract

Heavy metal contamination in river systems has become a cause for concern due to complex transport mechanisms. Sediment plays a significant role in the behaviour and fate of metals in aquatic ecosystems. The present study was carried out to investigate seasonal heavy metal contamination (As, Cr, Cu, Fe, and Zn) and the possible response of macrobenthos to wastewater effluent discharge along the longitudinal gradient of the Apies River. The bottom sediment samples were collected from ten (A-J) sites along the longitudinal gradient of the Apies River. Five (*n* = 5) samples were collected from each site using acid-pretreated bottles. Digestion was carried out using aqua regia, and metal analyses were performed using inductively coupled plasma optical emission spectroscopy (ICP-OES). Macrobenthos were collected through substrate disturbance and the use of a dip net. Sites A and B exhibited negligible to moderate metal concentrations, whereas Sites C—J showed considerable concentrations, with Cr and Cu exceeding the guidelines for aquatic ecosystems. No definite trend was observed for seasonal variation; however, sites immediately after wastewater plant discharge points showed high contamination, which suggests their possible role in metal contamination. Macrobenthos biomass showed a negative correlation with Cr and Cu, and with the contamination degree, pollution load index, and the potential risk index (RI). Heavy metal contamination indices indicated moderate to high contamination from Sites D—J, with the RI signifying low ecological risk. These findings provide an insight into the impact of wastewater effluents on metal enrichment, hence increasing the ecological risk.

**Supplementary Information:**

The online version contains supplementary material available at 10.1007/s10661-026-15348-4.

## Introduction

Heavy metal enrichment has been a cause for concern in river systems, particularly those with numerous point sources of pollution. Anthropogenic activities such as wastewater works, mining, agriculture, power stations, and industrial activities were found to drive metal pollution in river systems (Anh et al., 2023; Ćujić et al., 2016; Dash et al., 2021b). Heavy metals are persistent and tend to sink to the bottom sediment and remain fixed for a long period. However, variation in factors such as pH, salinity, temperature, hydrodynamics, redox potential, bioturbation, and sediment texture may result in metal resuspension back into the water column (Jia et al., 2021; Taka et al., 2022). According to Huang et al., (2020), sediment plays a significant role in the transportation and storage of metals. Moreover, Mohajane & Manjoro (2022) emphasised that sediment has the potential to serve as a source of heavy metals in the absence of external sources.

Numerous studies have explored the ecological risk of heavy metal contamination in sediment using various indices; however, the response of organisms associated with the sediment has been overlooked. These sediments provide sanctuary to a diverse range of macroinvertebrates, which form the base of the food chain (Guo et al., 2018). Moreover, the sediment-dwelling invertebrates, macrobenthos, are regarded as good and reliable bioindicators of heavy metal pollution due to their intolerance and susceptibility, long lifespan, and sedentary behaviour (Adesakin et al., 2023; Han & Han, 2024). According to Dash et al., (2021a), the sedentary behaviour of macrobenthos makes them reliable indicators of historical exposure to heavy metals. Therefore, integrating macrobenthos responses in ecological risk assessment has the potential to interpolate underlying mechanisms of toxicity in a river system.

A river is a longitudinal ecosystem, and the different geological and geomorphological characteristics in different stretches influence the dynamics of metal contamination (Hao et al., 2024; Macklin et al., 2006). Ecological risks of heavy metal contamination in freshwater systems have been fairly explored; however, little has been done on river systems with multiple sources along the longitudinal gradient and the association with macrobenthos biomass. The integration of ecological risk and the response of macrobenthos along the longitudinal gradient of a river with multiple sources of pollutants could provide insight into pollution modeling and the potential effect on other biota in freshwater ecosystems. In the present study, five heavy metals, arsenic (As), chromium (Cr), copper (Cu), iron (Fe), and zinc (Zn), were analysed in sediment from the Apies River in Tshwane, South Africa. The heavy metals were investigated for contamination enrichment, and their association with the macrobenthos assemblage was also explored. The three objectives of this study were to (1) explore the spatial–temporal distribution of heavy metals along the longitudinal gradient; (2) quantify heavy metal contamination and perform ecological risk assessment for concentration in sediment; (3) explore the association between the ecological risk index and macrobenthos biomass.

## Materials and methods

### Study areas

**Fig. 1:**
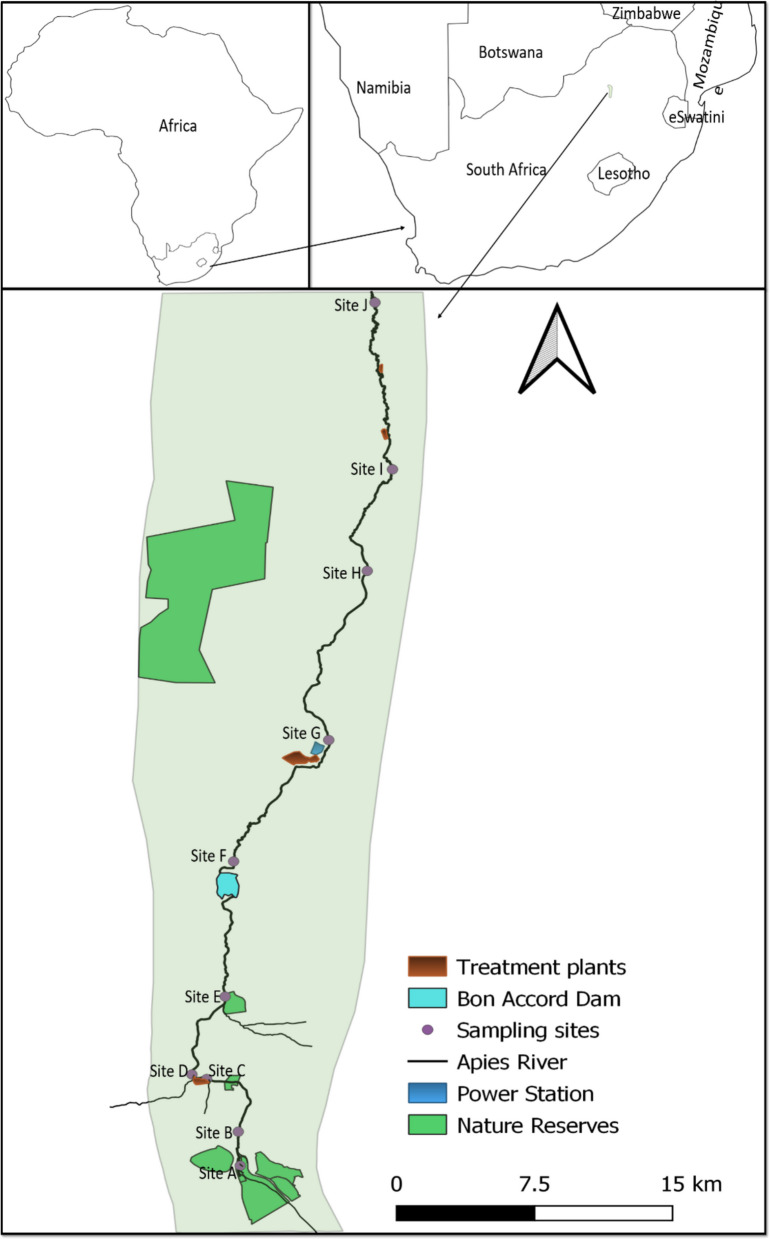
The Apies River catchment map showing all ten sampling sites

The Apies River originates from a nature reserve, south of Tshwane, and flows through the town, urban settlement, and then agricultural area (Fig. 1). The river receives effluents from three wastewater plants as it flows downstream, and considerable heavy metal concentrations have been reported in its surface water (Abia et al., 2018; Tau et al., 2021). Moreover, the river has been earmarked as a potential source of water for the agricultural park in the catchment (Le Roux et al., 2021).

### Macrobenthos sampling and biomass estimation

Macrobenthos were collected at 10 sites along the longitudinal gradient of the Apies River during dry and wet seasons in 2023 and 2024 sampling surveys. Sampling was performed following the Dickens & Graham (2002) SASS protocol, which was amended for macrobenthos. Five points were chosen at each site where an area of 1 m^2^ of sediment was disturbed, and the dip net was used to collect the dislodged macrobenthos. The points were of different depths, ranging from 20 to 600 cm, and randomly distributed at each site. The identification was performed using the Gerber & Gabriel (2002a, 2002b) identification guide and illustration. Macrobenthos were classified, counted, and left at 60℃ in a drying oven and weighed daily until no change was observed between days. Biomass was estimated following the Lovegrove (1966) and Guglielmo et al., (2002) protocol.

### Sediment sampling and metal analysis

Sediment samples were collected in each plot where macroinvertebrates were sampled (10 sites). Five samples were collected from each 1 m^2^ plot to make a composite for that particular plot, and hence, 5 samples for each site. and stored in acid-pretreated bottles. Samples were kept in a cooler box and later transferred to a freezer until analysis. Analysis was carried out following the method described in Misra et al., (2024) and Lebepe et al., (2024). Samples were oven-dried at 60℃ and ground using a mortar and pestle. The aqua regia, 3-hydrochloric acid (HCl):1-nitric acid (HNO_3_), complemented with 30% hydrogen peroxide (H_2_O_2_), was used for digestion. Samples were filtered and brought to the 100 ml mark using distilled water. Metal analysis was conducted using inductively coupled plasma optical emission spectroscopy (ICP-OES). Sediment guidelines, threshold effect level (TEL), which is the concentration below which adverse effects are not probable, and the probable effect level (PEL), which is the concentration above which adverse effects are probable (MacDonald et al., 2000), were used.

### Quality control

For quality control, samples were digested with blanks, and the DORM-4 certified reference materials (CRMs) for fish supplied by the Canadian National Research Council (CNRC) were also used during analysis. The recovery ranged from 91–102%. All chemicals used were of analytical grade (Merck®).

### Metal contamination indices

#### Contamination factor

The contamination factor (CF) is the ratio of the concentration of a single heavy metal in sediment to its background concentration, and it was calculated using Eq. 1 as per Hakanson (1980).

1 $$\mathrm{CF}=\frac{\mathrm{Cn}}{\mathrm{Bn}}$$

where C_n_ is the concentration in the sample and B_n_ is the background concentration. The background concentration from Turekian & Wedepohl (1961) was used. The classification categories described by El-Amier et al., (2017) were used to classify the contamination factor, which ranged from < 1 for a low contamination factor to ≥ 6 for a very high contamination factor.

#### Enrichment factor

Enrichment factor (EF) is also defined as the ratio of the concentration of a single heavy metal in sediment to its background concentration; however, with the EF, conservative heavy metals that are not easily influenced by weathering, such as aluminium (Al) or iron (Fe), are integrated to ascertain the contamination. In the present study, Fe was used as the conservative heavy metal, and the EF was calculated using Eq. 2.

2 $$\mathrm{EF}= \frac{({{\mathrm{C}}_{\mathrm{n}}/{\mathrm{C}}_{\mathrm{Fe}})}_{\mathrm{sample}}}{({{\mathrm{B}}_{\mathrm{n}}/{\mathrm{B}}_{\mathrm{Fe}})}_{\mathrm{Background}}}$$

#### Geoaccumulation index

The geoaccumulation index (I_geo_) determines the pollution level for each heavy metal of interest and is described as the ratio of heavy metal concentration in the sediment sample to the background concentration existing in a natural environment (Custodio et al., 2024; Turekian & Wedepohl, 1961). The index was calculated using Eq. 3 as per Turekian & Wedepohl (1961).

3 $${\mathrm{I}}_{\mathrm{geo}}={\mathrm{log}}_{2}\left[\frac{{\mathrm{C}}_{\mathrm{n}}}{{1.5\mathrm{B}}_{\mathrm{n}}}\right]$$

where 1.5 is the coefficient for minimising the impact of the background concentration due to lithological variation, and B_n_ is the background concentration existing in a natural environment as per Turekian & Wedepohl (1961). Muller (1969) classification categories, as described by Addo-Bediako et al., (2021), were used to categorise pollution level, which ranged from 0 for unpolluted to ≥ 5 for extremely polluted.

#### Pollution load index, degree of contamination

The pollution load index (PLI) determines the extent of sediment pollution by metals and their environmental impact (Custodio et al., 2024). This index was calculated using Eq. 4 as per Tomlinson et al., (1980).

4 $$\mathrm{PLI}= {\left({\mathrm{CF}}_{1}\times {\mathrm{CF}}_{2}\times {\mathrm{CF}}_{3}\dots \dots ..{\mathrm{CF}}_{\mathrm{n}}\right)}^{\frac{1}{\mathrm{n}}}$$

where CF_n_ is the contamination factor for each heavy metal. The PLI of < 1 denotes an unpolluted site, whereas PLI > 1 denotes a polluted site. The sum of CF, which is the contamination degree (CD), was calculated as per Hakanson (1980) using Eq. 5.

5 $$\mathrm{CD}= {\sum }_{\mathrm{i}=1}^{\mathrm{i}=\mathrm{n}}\mathrm{CF}$$

#### Ecological risk assessment

The potential ecological risk index (RI) is used to assess the overall degree of contamination or ecological risk of metals in sediment. It is described as the sum of ecological risk factors (E_r_) for each metal of interest. The E_r_ is calculated using the toxicity coefficient and CF of each metal (Eq. 6). The toxicity coefficients used for this study were 7, 10, 2, 5, 1, and 1 for Sb, As, Cr, Cu, Fe, and Zn, respectively (Devanesan et al., 2017; Hakanson, 1980). The RI was calculated using Eq. 7.

6 $${\mathrm{E}}_{\mathrm{r}}^{\mathrm{i}}= {\mathrm{T}}_{\mathrm{r}}^{\mathrm{i}} \times {\mathrm{CF}}_{\mathrm{r}}^{\mathrm{i}}$$

7 $$Ri= {\sum }_{i=1}^{n}{E}_{r}^{i}$$

where T_r_ is the toxicity coefficient, E_r_ is the ecological risk factor for each metal, and RI is the potential ecological risk index for the site.

### Data analysis

All statistical analyses were performed using R software. Spatial variation of metal concentrations in sediment was compared using analysis of variance (ANOVA). The residuals were tested for normality and homogeneity of variance using the Kolmogorov–Smirnov test and Levene’s test. The posthoc test was carried out to determine the sites that showed a significant difference. Seasonal variation was tested using an independent-sample t-test. The data normality and the homogeneity of variance were tested using the Kolmogorov–Smirnov test and Levene’s test. The correlation was tested using the chart.Correlation function of the “PerformanceAnalytics” package in R (Peterson et al., 2020), whereas principal component analysis was run using the fviz_pca_biplot function in the “ade4” and “factoextra” packages. The significance was set at p < 0.05.

## Results and discussion

### Spatial distribution of metals

**Fig. 2:**
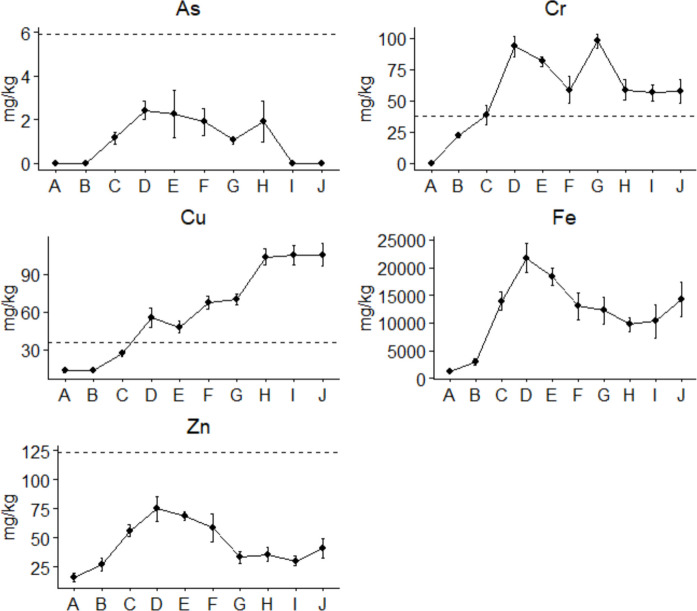
Heavy metal concentration observed at 10 sites along the Apies River during 2023–2024 surveys, with the abline denoting the guideline for aquatic ecosystems

Sediment is known as a sink for metals in aquatic ecosystems (Custodio et al., 2024). In the present study, significant metal concentrations were observed across sites (Fig. 2, Table 1). No definite trend was observed for metal concentration between sites; however, Sites A and B exhibited lower concentrations for all metals (Fig. 2, Table 1), confirming their pristine condition, as they are located in the headwaters. Nevertheless, most metals showed significantly higher concentrations from Site C, which is located downstream of the Tshwane town, before the wastewater effluent discharge point (Fig. 1), suggesting the impervious catchment as the driver for the increase. Wu et al., (2025) emphasized that commercial activities and traffic pollution are primary drivers of pollution in urban stretches of river systems. Site D showed a further increase compared to Site C, with an indefinite trend being observed from Site E to J (Fig. 2). Site D is located approximately 1 km downstream of the wastewater work discharge point (Fig. 1), suggesting that the wastewater effluents might have driven an increase in heavy metal concentrations at this site.

**Table 1:** Mean concentrations of heavy metals observed at sampling sites in the Apies River during the wet and dry seasons of the 2023–2024

Sites	Seasons	As	Cr	Cu	Fe	Zn
Site A	Wet	< 0.001	< 0.001	12.74 ± 1.53	1148.75 ± 110.38	13.05 ± 0.94
	Dry	< 0.001	< 0.001	13.71 ± 0.69	1337.12 ± 76.76	19.43 ± 1.26
Site B	Wet	< 0.001	21.05 ± 2.04	12.94 ± 0.69	2548.26 ± 133.68	22.15 ± 1.87
	Dry	< 0.001	22.76 ± 0.94	13.55 ± 1.12	3470.18 ± 527.42	33.14 ± 1.52
Site C	Wet	1.05 ± 0.28	31.15 ± 1.23	28.45 ± 2.42	14438.02 ± 1598.70	58.90 ± 3.48
	Dry	1.20 ± 0.26	**45.46 ± 2.99**	26.05 ± 2.92	13394.13 ± 1742.30	53.28 ± 4.64
Site D	Wet	2.32 ± 0.41	**87.04 ± 5.33**	**48.70 ± 1.93**	20204.70 ± 2453.20	65.74 ± 4.51
	Dry	2.51 ± 0.45	**99.81 ± 3.89**	**62.50 ± 3.29**	23204.62 ± 1902.50	83.71 ± 4.63
Site E	Wet	3.25 ± 0.88	**84.07 ± 3.39**	**44.93 ± 2.57**	19510.45 ± 1049.60	70.42 ± 2.25
	Dry	1.39 ± 0.25	**79.17 ± 2.62**	**51.45 ± 3.41**	17307.45 ± 1207.80	67.04 ± 4.56
Site F	Wet	2.31 ± 0.49	**68.11 ± 4.28**	**70.42 ± 4.77**	12490.37 ± 2238.20	69.02 ± 5.90
	Dry	1.49 ± 0.46	**49.11 ± 5.24**	**64.19 ± 3.41**	13597.16 ± 2700.20	48.07 ± 4.78
Site G	Wet	1.05 ± 0.22	**101.26 ± 3.29**	**70.93 ± 4.71**	13108.43 ± 2604.30	37.05 ± 1.64
	Dry	1.05 ± 0.15	**94.17 ± 4.37**	**69.14 ± 4.15**	11370.38 ± 2282.20	29.48 ± 4.27
Site H	Wet	2.75 ± 0.53	**62.07 ± 9.57**	**99.04 ± 3.42**	10406.75 ± 1468.10	31.90 ± 3.32
	Dry	1.09 ± 0.10	**55.42 ± 6.32**	**108.32 ± 5.19**	9023.41 ± 6411.20	39.71 ± 4.83
Site I	Wet	< 0.001	**52.80 ± 4.92**	**100.47 ± 5.94**	8307.38 ± 964.07	27.17 ± 2.49
	Dry	< 0.001	**59.81 ± 5.64**	**110.18 ± 5.92**	12370.16 ± 3095.90	33.41 ± 3.72
Site J	Wet	< 0.001	**52.07 ± 3.84**	**107.92 ± 7.24**	13281.05 ± 2964.90	42.19 ± 7.43
	Dry	< 0.001	**62.36 ± 10.89**	**102.70 ± 10.68**	15290.12 ± 3404.20	39.71 ± 10.49
**Guidelines (TEL)**		5.90	37.30	35.70	-	123
**Guidelines (PEL)**		17	90	197	-	315

Bold: Exceeded quality guidelines.

*TEL* Threshold effect level

*PEL* Probable effect level

There was a significant difference for As concentrations (F = 6.97, *p* < 0.05), with Sites D, E, F and H showing higher concentrations compared to other sites (Fig. 2). Arsenic concentration was below the detection level at Sites A, B, I and H, with notable concentrations being observed for other sites. However, As concentrations were below the CCME (2001) of 5.90 mg/kg across all sites. Similarly, Cr showed a significant difference between sites (F = 90.12, *p* < 0.05), with Sites D, E, and G exhibiting higher concentrations compared to other sites. Chromium concentration below the detection limit was observed at Site A, with a notable concentration, which was still below the CCME (2001) guideline, being observed at Site B. In contrast, other sites showed Cr concentrations exceeding the guideline, with Site G showing concentrations above the MacDonald et al., (2000) PEL (Table 1).

Interestingly, Cu showed an increasing trend from Site A to J (F = 415,70, *p* < 0.05) (Fig. 2), with Sites A to C showing concentration below the CCME (2001) of 35.70 mg/kg, and Sites D to J exhibiting concentration exceeding the guideline (Table 1). Iron showed an increasing trend from Site A to D, decreased from Site E to H, and then increased again from Site I to J (F = 85.18, *p* < 0.05). There is no guideline for Fe from the literature; however, a higher concentration was observed at Site D, with Site A showing the least (Fig. 2). Similarly, Zn showed an increasing trend from Site A to D, then decreased until Site G (F = 76.32, *p* < 0.05); however, no significant change was observed from Site G to J (*p* > 0.05) (Fig. 2). Despite other metals exhibiting concentrations above the guidelines across most sites, Zn was within the CCME (2001) guideline across all sites, suggesting that the wastewater works along the Apies River have not significantly increased its concentration.

Generally, metals started showing hazardous concentrations from Site C, with Site D and other downstream sites exhibiting further increases. Site C reflects the cumulative contamination from the entire Tshwane town. Impervious catchments are known to influence the hydrologic regime of urban rivers through runoffs (Sohn et al., 2020), which wash contaminants into the river, affecting the water and sediment chemistry (Anh et al., 2023; Müller et al., 2020). In the present study, most metals were within the guidelines at Site C, with the exception of Cr. The Cr concentration observed at Site C was comparable to those reported by Ali et al., (2022) and Shammi et al., (2024), and lower than those reported by Wu et al., (2025) and Byrne et al. (2017) in urban rivers. Moreover, Mokgohloa et al., (2022) reported Cr concentration lower than that observed at Site D in natural rivers. The findings of the present study suggest that the urban catchment significantly increased Cr concentration at Site C, with other metals also showing considerable levels. Moreover, Site C provides a clear picture of the potential impact of urban activities on metal enrichment in rivers, which becomes a cause for concern, particularly given the potential of urban rivers to harbour biota.

Site D is located immediately after the discharge point of the wastewater plants, and it showed an increase in metal concentrations compared to Site C, whereas Site E, which is located further downstream, showed no difference compared to Site D. Sites D and E exhibited Cr and Cu concentrations exceeding the guidelines. Wastewater effluents are known to drive metal increases in river systems (Dwivedi et al., 2018). Moreover, Mokarram et al., (2020) observed increased concentration of metals after the wastewater effluent discharge point. Similarly, Kumar et al., (2023) reported increased metal concentration as a result of wastewater effluents. Although the Tshwane town contributed to the metal increase at Sites D and E, it is evident that wastewater effluents further increased the concentrations. Nevertheless, a significant decrease in Cr, Fe, and Zn was reported at Site F compared to Site E. Site F is located immediately after the Bon Accord Dam. According to Wang et al., (2019), impoundments in rivers allow metals to settle to the bottom sediment. Moreover, other studies reported a decrease in pollutants immediately after impoundments in river systems (Shimizu et al., 2020; Zhao et al., 2021). Despite other metals remaining constant, the role of Bon Accord Dam in sinking Cr, Fe and Zn may not be dismissed.

Site G is located immediately after another wastewater discharge point (Fig. 1). Despite Site G’s location, only Cr has shown a significant increase compared to Site F. In contrast, Cu showed no significant change from Site F to Site G, whereas a significant increase was observed at Sites H, I and J. Iron showed no significant increase as a result of wastewater discharge, and the concentrations decreased at Sites H and I, with an increase being observed at Site J, which was downstream of another wastewater effluent discharge point. From Site G, heavy metal concentrations showed no definite trend in their response to wastewater effluent discharge. Nevertheless, the general trend shows an increase at Site J, which is after two wastewater plants. Dwivedi et al., (2018) reported that industrial activities, urban wastewater effluents and agricultural activities are contributing immensely to metal contamination, whereas Kumar et al., (2023) observed high metal concentrations in the sediment of rivers receiving effluents from wastewater plants. Despite wastewater effluents showing to drive metal contamination in rivers, the adsorption capacity of sediment, hydrolysis and metal solubility, mobility, bioavailability, precipitation, and cation dynamics underlie the mechanism (Aradpour et al., 2020; Custodio et al., 2024). Moreover, the potential risk would also depend on the concentrations and the physical factors such as pH, salinity, redox potential, etc. (Zhang et al., 2021). Nevertheless, the constant discharge of wastewater effluents along the longitudinal gradient of the Apies River seems to generally increase metal concentrations in sediment.

### Seasonal trend of metal concentrations

Metals in sediment are expected to be higher during low-flow seasons due to reduced water velocity and quantity (Bhuyan & Bakar, 2017); however, this was not the case in the present study. Sites A and B showed relatively higher Zn concentrations during the dry season (p < 0.05), whereas Site C exhibited a high concentration of Cr during the dry season (Table 1). Similarly, Site D showed relatively higher concentrations for Cr, Cu and Fe during the wet season and Zn during the dry season (*p* < 0.05). Nevertheless, these findings were comparable to those observed in other related studies (Duncan et al., 2018; Huang & Gergel, 2022). In contrast, Site E showed relatively higher concentrations of As and Fe during the wet season compared to the dry season (Table 1). A similar trend was observed at Site F, where As, Cr and Zn exhibited higher concentrations during the wet season compared to the dry season (Table 1). Moreover, Site G showed higher concentrations of Fe and Zn during the wet season compared to the dry season. Nevertheless, Site H showed higher As concentrations during the wet season and a higher Zn concentration during the dry season, whereas Sites I and J exhibited a higher Fe concentration during the dry season (Table 1).

Lundy et al., (2017) and Pandey & Singh (2017) observed higher metal concentrations during the wet season compared to the dry season, whereas Saifullah et al., (2025) reported higher concentrations of Cu, Cr, and Zn during the dry season, and Fe during the wet season. The observed trends suggest that heavy metals exhibit different behaviour in different conditions, primarily the temperature. Moreover, flow velocity, sediment texture, pH, etc., are known to drive metal settlement (He et al., 2023). The adsorption capacity of sediment, hydrolysis, and metal solubility, mobility, bioavailability, precipitation, and cation dynamics may also underlie the settlement mechanism for each heavy metal (Aradpour et al., 2020; Custodio et al., 2024), which could be the explanation for the observed trend along the longitudinal gradient of the Apies River. According to Mukwevho et al., (2025), high metal concentration during the wet season may be associated with increased surface runoff and erosion from urban and industrial areas. The indefinite trend of seasonal variation of heavy metals may also suggest the existence of chronic anthropogenic activities and hydrologic shifts that result in cyclical and unpredictable patterns. Moreover, the trend ultimately results in uncertainties when employing pollution modeling to predict metal behaviour in a longitudinal ecosystem.

### Metal contamination indices and ecological risk assessment

**Fig. 3:**
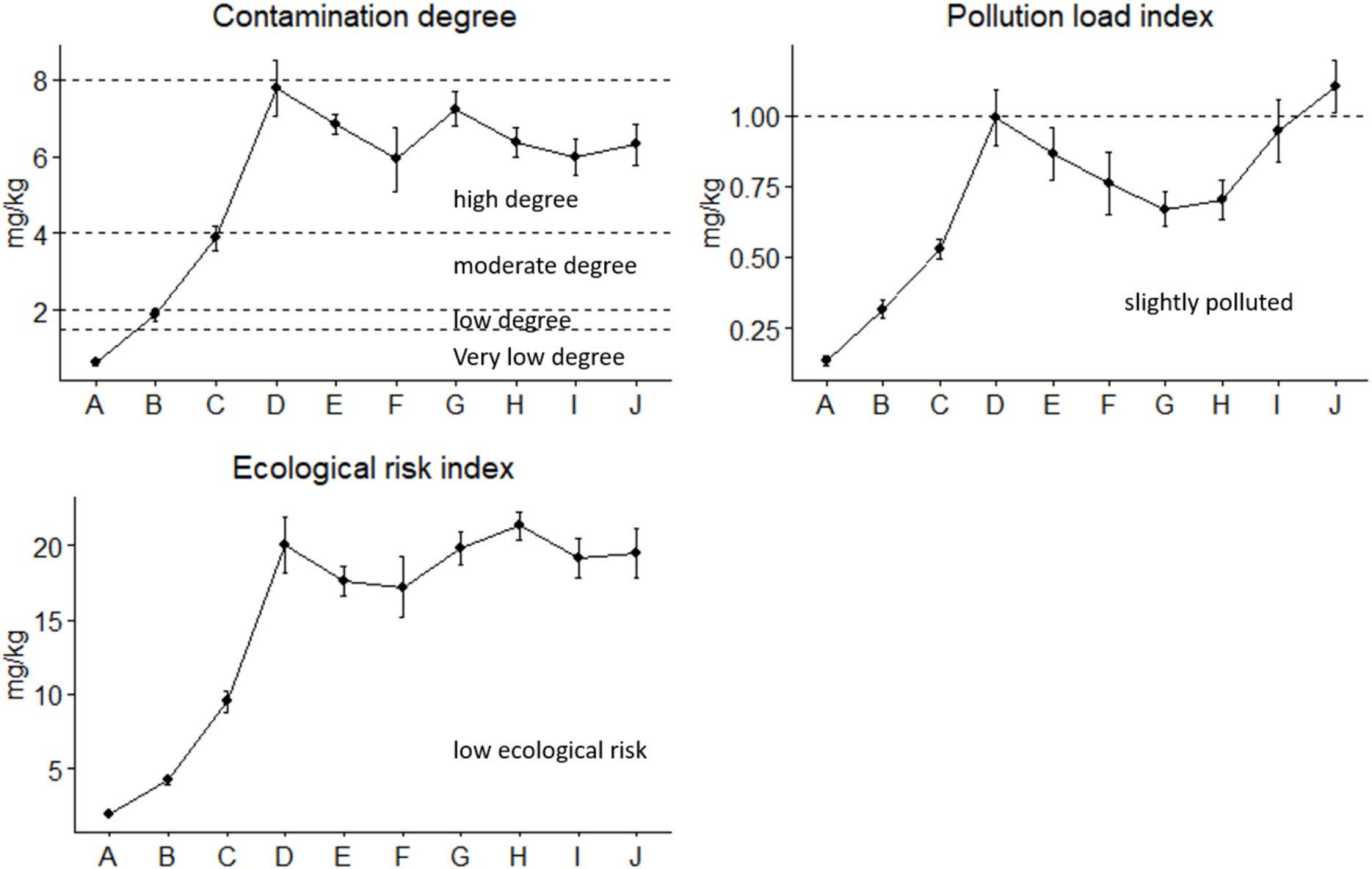
Heavy metal contamination indices and ecological risk index observed at 10 sampling sites along the Apies River during 2023–2024 surveys

The results on the heavy metal pollution indices are reported in Table 2 and Fig. 3. Pollution indices were used to classify contamination levels as per Muller (1969), Tomlinson et al., (1980), Weissmannová & Pavlovský (2017), and Liao et al., (2022) (Table S1). In the present study, the CF ranged from 0.11 to 4.69 across sites, with Cr and Cu being heavy metals of concern (Table 2). Arsenic exhibited a low contamination category across all sites, whereas Cr showed considerable contamination for Sites D and G, with other sites except A and B showing considerable contamination (Table 1 & Table S1). However, Cu showed moderate contamination from Site D to J, with Sites H, I, and J exhibiting a CF > 2 (Table 2 & Table S1). Iron exhibited low contamination, whereas Zn showed a CF < 1 except for Sites C to F. The findings of the present study are comparable to those observed by other related studies (Ncayiyana et al., 2025; Wu et al., 2025) and higher than those observed by Zheng et al., (2024). Moreover, Mohajane & Manjoro (2022) reported moderate to considerable contamination for Cr, Cu, and Zn in a river impacted by wastewater effluents. Sites D and G commonly exhibited higher CF compared to other sites for most heavy metals, affirming the role of wastewater works as a driver for this metal contamination.

**Table 2:** Heavy metal contamination indices observed at different sites in the Apies River during the wet and dry seasons in 2023–2024 surveys

Sites	Seasons	Contamination factor					Enrichment factor					Geoaccumulation index				
		As	Cr	Cu	Fe	Zn	As	Cr	Cu	Fe	Zn	As	Cr	Cu	Fe	Zn
Site A	Wet			0.32	0.02	0.23			4.77	1.00	3.49			−2.24	−5.95	−2.69
	Dry			0.34	0.03	0.35			4.85	1.00	4.91			−2.13	−5.73	−2.12
Site B	Wet		0.97	0.32	0.05	0.40		8.30	6.01	1.00	7.34		−0.63	−2.21	−4.80	−1.93
	Dry		1.04	0.34	0.07	0.57		8.21	4.69	1.00	7.95		−0.53	−2.15	−4.36	−1.39
Site C	Wet	0.11	1.44	0.71	0.31	1.05	0.38	4.77	2.36	1.00	3.46	−3.76	−0.06	−1.08	−2.30	−0.51
	Dry	0.13	2.10	0.65	0.28	0.95	0.48	7.48	2.33	1.00	3.38	−3.51	0.49	−1.21	−2.41	−0.66
Site D	Wet	0.25	4.03	1.22	0.43	1.18	0.59	9.53	2.88	1.00	2.77	−2.60	1.42	−0.30	−1.82	−0.35
	Dry	0.27	4.62	1.56	0.49	1.50	0.56	9.46	3.19	1.00	3.07	−2.49	1.62	0.06	−1.61	−0.01
Site E	Wet	0.34	3.89	1.12	0.41	1.26	0.83	9.43	2.71	1.00	3.05	−2.20	1.37	−0.43	−1.86	−0.25
	Dry	0.15	3.67	1.29	0.37	1.20	0.41	10.02	3.53	1.00	3.27	−3.34	1.29	−0.23	−2.04	−0.33
Site F	Wet	0.25	3.15	1.76	0.26	1.23	0.96	12.15	6.86	1.00	4.73	−2.62	1.07	0.23	−2.52	−0.29
	Dry	0.16	2.27	1.60	0.29	0.86	0.57	8.01	5.72	1.00	3.09	−3.27	0.59	0.10	−2.40	−0.81
Site G	Wet	0.11	4.69	1.77	0.28	0.66	0.41	17.38	6.55	1.00	2.46	−3.75	1.64	0.24	−2.46	−1.18
	Dry	0.11	4.36	1.73	0.24	0.53	0.48	18.49	7.32	1.00	2.20	−3.75	1.54	0.20	−2.66	−1.52
Site H	Wet	0.30	2.87	2.48	0.22	0.57	1.38	13.17	11.41	1.00	2.61	−2.36	0.92	0.72	−2.78	−1.40
	Dry	0.12	2.57	2.71	0.19	0.71	0.62	13.44	14.27	1.00	3.72	−3.67	0.77	0.85	−2.98	−1.09
Site I	Wet		2.44	2.51	0.18	0.48		13.98	14.41	1.00	2.79		0.70	0.74	−3.10	−1.63
	Dry		2.77	2.75	0.26	0.60		11.07	11.04	1.00	2.43		0.88	0.88	−2.55	−1.34
Site J	Wet		2.41	2.70	0.28	0.75		8.91	9.91	1.00	2.70		0.68	0.84	−2.44	−1.01
	Dry		2.89	2.57	0.32	0.71		9.11	8.14	1.00	2.33		0.93	0.77	−2.24	−1.13

The EF ranged from 0.38 to 18.49 across sites, with Cr and Cu being the heavy metals of concern (Table 2). Chromium showed significant enrichment from Site D to J, whereas Cu exhibited significant enrichment from Site F to J, suggesting the wastewater effluents as potential drivers. Yan et al., (2025) reported an EF ranging from 0.8 to 8.1 for Cr and 1.7 to 28.9 for Cu in a river impacted by wastewater effluents. Moreover, Kola et al., (2025) reported low to significant enrichment for Cr and Cu in a wetland impacted by wastewater effluents. Zinc exhibited moderate enrichment across all sites. The EF was calculated using Fe as the conservative metal, due to its resilience to anthropogenic stressors (Pandey & Singh, 2017). However, its concentration influenced the EF, particularly in Sites A and B. Nevertheless, Kolawole Tesleem et al., (2018) emphasised that an EF > 1 may be an indication of additional anthropogenic stressors in the systems.

Corroborating trends observed for CF and EF, the I_geo_ categorised the As contamination as uncontaminated across all sites, moderate contamination being observed for Cr from Site D to G. Moderate contamination was also observed for Cu at Sites D and F to J (Table 1 & Table S1). These findings are comparable to those observed by Kola et al., (2025) and Fadlillah et al., (2023) in rivers impacted by urban and industrial wastewater effluents. Kolawole Tesleem et al., (2018) reported I_geo_ < 0 for most metals, with Cr and Cu exhibiting I_geo_ > 0 in a river impacted by domestic and industrial wastewater effluents. There is no clear trend on the I_geo_ along the river system; however, Sites D and G, which are located a few kilometers downstream of the wastewater plants, exhibited significant index values. The I_geo_ and EF are usually showing some discrepancies with regard to pollution status characterisation due to different choices of reference elements or background concentrations (Abdullah et al., 2020). Nevertheless, the two are still among the most reliable indices for assessing sediment contamination.

Corroborating the afore-mentioned contamination indices, CD, which is described as a cumulative environmental pollution index that represents the sum of CFs (Dash et al., 2021b), has also exhibited a high degree of contamination for Sites C to J, with Sites D and G showing higher index values (Fig. 3, Table S1). The observed CD values are comparable to those reported by Kola et al., (2025) in wastewater effluent-polluted rivers and lower than those reported by Saifullah et al., (2025) in a river impacted by domestic wastes. Contrast the spatial CD trend, the higher PLI was observed at Sites D and J (PLI ≥ 1), categorising them as polluted, whereas Sites C and E to I exhibited PLI > 0.5, which still categorised them as non-pristine (Fig. 3). Despite this contrasting trend, the two indices exhibited a similar decreasing trend from Site D to F (Fig. 3), which suggests self-cleansing capacity and dilution of metals as the distance increases from the primary pollution source.

Despite some indices exhibiting moderate and significant contamination in relation to wastewater effluent discharge, the RI remained low, with Cr and Cu being the primary contributors across all sites. Nevertheless, Effendi et al., (2016) and Kumar et al., (2023) found Cr and Cu to be the least contributors to the RI in rivers impacted by wastewater effluents and agriculture. Saifullah et al., (2025) reported low RI for metal contamination in a river impacted by wastewater effluents, an agro-fertilizer factory, and power plants. In the present study, the immediate sites downstream of the wastewater effluent discharge points exhibited moderate to high contamination for heavy metals, particularly Cr and Cu. Nevertheless, the spatial distribution in relation to the main sources remains obscure due to the dynamics of longitudinal ecosystems. The dilution and assimilation capacity are among the factors influencing the behaviour of a river system. These phenomena could be the possible explanation for the observed spatial trend in the Apies River.

Metal contamination showed to have increased compared to background levels, which were also supported by the CF, EF, I_geo_, CD, and PLI. Nevertheless, the overall RI based on the background concentrations used in the present study indicated low ecological risk across all sites. It is imperative to develop local background values due to the association between these values and the geology of the area (Liao et al., 2022). It is worth noting that the RI was calculated based on 5 metals. Moreover, most of these metals were also below the PEL stipulated by MacDonald et al., (2000), with only Cr being of particular concern.

### Correlation between metals

**Fig. 4:**
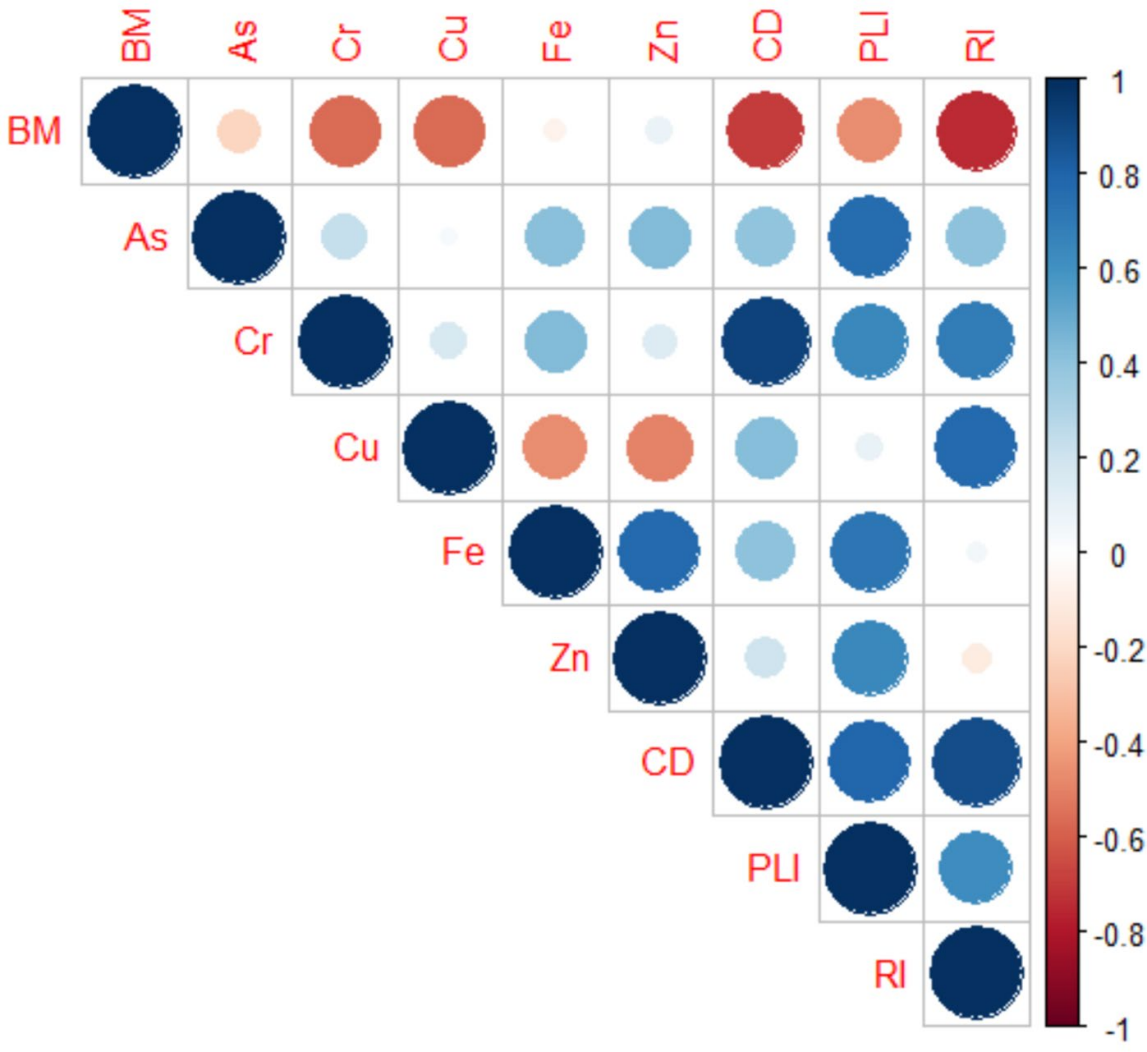
Correlation for metals and macrobenthos biomass observed in the sediment of the Apies River during 2023–2024 surveys. Note: The data for all sites and both seasons were pooled

**Fig. 5:**
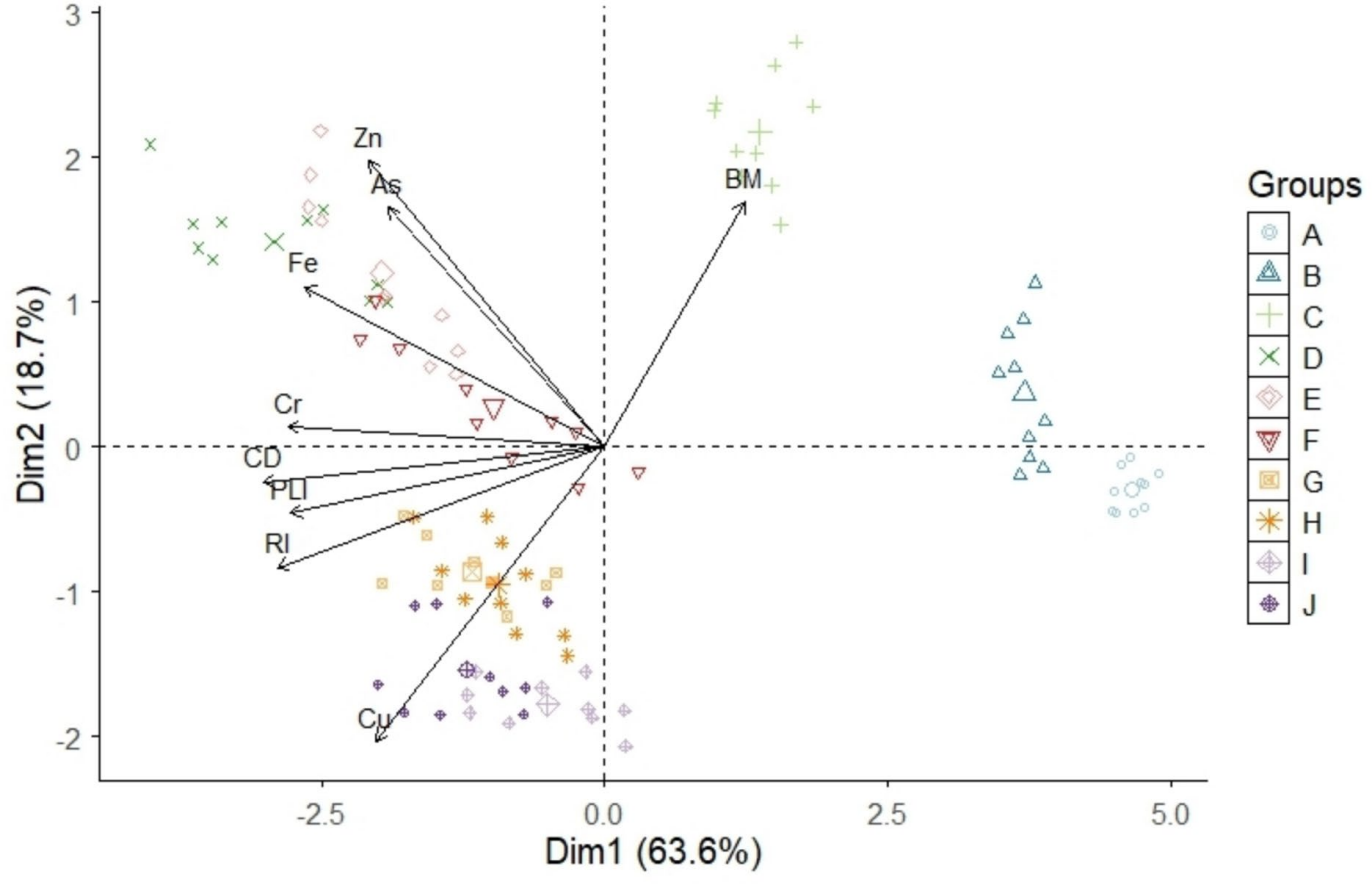
Principal component analysis for heavy metals in sediment, risk indices and biomass across 10 sampling sites

For correlation analysis, sites were pooled to get an overall association between metals. Generally, poor to strong inter-metal correlations were observed in the present study. Relationships were observed for As-Fe (r = 0.42, *p* < 0.05), As-Zn (r = 0.43, *p* < 0.05), Cr-Fe (r = 0.43, *p* < 0.05), Cu-Fe (r = −0.47, *p* < 0.05), Cu–Zn (r = 0.49, *p* < 0.05) and Fe-Zn (r = 0.78, *p* < 0.05) (Figs. 4&5). Inter-metal relationships may be used to determine potential sources; however, the complexity of the system may influence the dynamics of most metals, particularly in river systems characterized by different land use along their longitudinal gradient (Rajasekar et al., 2024). However, the strong correlation between Fe and Zn may still suggests common source.

### Macrobenthos response and association with metal contamination

Macrobenthos observed during this study included Gomphidae, Oligochaeta, Hirudinea, Chironomidae, Simulidae, Corbiculidae, Leptophlebiidae, and some caddisflies. However, there were no definite trends in their abundance along the longitudinal gradient, particularly in relation to contaminant source areas. Sites A and B were dominated by intolerant taxa such as Leptophlebiidae and some caddisflies. According to Brand & Miserendino (2011) and Farooq et al., (2021), Leptophlebiidae and caddisflies are indicators of good-quality water. Site C is located immediately after Tshwane town; therefore, its condition reflects a cumulative impact of the entire impervious catchment. This site was also characterised by high biomass of macrobenthos (Fig. 5), which included Simulidae and Chironomidae. However, Site G exhibited a higher abundance of highly tolerant Oligochaeta and Hirudinea, implicating the wastewater effluents and runoff from agricultural areas as the possible drivers. These findings were comparable to those observed in numerous studies where tolerant taxa were associated with contaminated sites (Adesakin et al., 2023; Kebede et al., 2020; Rico-Sánchez et al., 2022). Literature has shown that Oligochaeta, Hirudinea, Chironomidae, Simulidae and Corbiculidae are tolerant taxa associated with contaminated waters (Aristone et al., 2022; Gao et al., 2023; Lebepe et al., 2022). The contamination levels observed at different sites showed no seasonal variation, which was also supported by the biomass of macrobenthos.

The biomass showed a negative association with Cr (r = −0.57, p < 0.05) and Cu (r = −0.57, p < 0.05) concentrations, which were corroborated by a negative correlation between the biomass and the three contamination indices, CD (r = −0.69, p < 0.05), RI (r −0.75, p < 0.05) and PLI (r = −0.47, p < 0.05) (Fig. 4). These metal contamination and macrobenthos relationships were comparable to those observed by Liang et al., (2024) and Bian et al., (2016). Moreover, Dong et al., (2021) and Hu et al., (2019) found the structure of macrobenthic communities and biomass changing with increasing metal pollution. Moreover, Zhao et al., (2023) also reported a negative association between macrobenthos biomass and sediment contamination. In contrast, Zhang et al., (2021) reported no association between high copper concentration and macrobenthos biomass. Sediment is known as a potential stressor for aquatic ecosystems, as it provides a matrix for contaminant settlement (Custodio et al., 2024). However, the bioavailability of contaminants such as metals in sediment may be influenced by numerous physical factors, such as water pH, temperature, and salinity, sediment texture, etc. (He et al., 2023), which ultimately drives the response of macrobenthos. Nevertheless, the continuous discharge of effluents along the longitudinal gradient of the Apies River system may underlie the dynamics of metal bioavailability and the partitioning between the sediment and water column, which may complicate the ecological risk modelling.

## Limitations

Macrobenthos were identified to family levels and the sediment grain size was also not determined.

## Conclusion

The study assessed the ecological risk of heavy metal contamination and the response of macrobenthos along the longitudinal gradient of the Apies River. Sediment was found to be contaminated, with different sites exhibiting varying concentrations based on its location relative to primary sources, which included urban activities and wastewater effluents. The contamination status of Sites D—J ranged from moderately to significantly/highly contaminated, whereas the potential ecological risk remained low. Chromium and Cu were found to be elements of concern, particularly at Sites D and G. Moreover, the two heavy metals showed a negative correlation with macrobenthos biomass, which also correlated negatively with CD, PLI, and RI. Despite Side D to J showing a clear spatial trend regarding metal contamination, no definite trend observed for seasonal variation across sites. Agricultural activities serve as additional stressors for Sites F to J. Generally, metal contamination indices were irrefutable in distinguishing pollution statuses in different sites, with those immediately after wastewater discharge points showing increased concentrations. These findings provide an insight into the potential role of urbanisation, wastewater effluents and agricultural activities in metal contamination, and the dynamics underlying the dilution and assimilation capacity of rivers. Modelling of the volume of effluents released by different wastewater plants into the Apies River is recommended to allow a comprehensive prediction of the potential impact of these plants on metal enrichment.

## Supplementary Information

Below is the link to the electronic supplementary material.

Supplementary file1 (DOCX 44 KB)

## Data Availability

Data is available upon request.

## Declarations

All authors have read, understood, and complied as applicable with the statement on “Ethical responsibilities of Authors” as found in the Instructions for Authors.
