# Supplementary material for: Ecological risk assessment of heavy metal contamination and macrobenthos response in the Apies River, South Africa
Source: Environ Monit Assess. 2026 May 4;198(5):546. doi: 10.1007/s10661-026-15348-4 (PMC13139275; doi:10.1007/s10661-026-15348-4)
Supplement: Supplementary file 1 — Supplementary file1 (DOCX 44 KB) [file 10661_2026_15348_MOESM1_ESM.docx]

**Ecological risk assessment of trace element contamination and macrobenthos response in the Apies River, South Africa**

Jeffrey Lebepe^1*^, Moleseng Claude Moshobane^1,2^, Mapurunyane Callies Selala^1^

*^1^Department of Biology and Environmental Sciences, Sefako Makgatho Health Sciences University, Pretoria 0204, South Africa. emails:* [*jlebepe@yahoo.com*](mailto:jlebepe@yahoo.com)*;* [*Callies.Selala@smu.ac.za*](mailto:Callies.Selala@smu.ac.za)

*^2^South African National Biodiversity Institute, Pretoria National Botanical Garden, Brummeria, Silverton, 0184 South Africa. email:* [*moshobanemc@gmail.com*](mailto:moshobanemc@gmail.com)

** Corresponding author’s e-mail:* [*jlebepe@yahoo.com*](mailto:jlebepe@yahoo.com)

**Table S1.** Criteria for categorizing contamination level for different indices (Muller, 1969; Weissmannová and Pavlovský, 2017; Liao et al., 2022; Tomlinson et al., 1980)

| **Indices** | **Levels** | **Contamination status** |
| --- | --- | --- |
| Enrichment Factor | EF ≤ 1 | Minimal contamination |
|  | 1 < EF ≤ 5 | Moderate enrichment |
|  | 5 < EF ≤ 20 | Significant enrichment |
|  | 20 < EF ≤ 40 | Very high enrichment |
|  | 40 < EF | Extremely high enrichment |
|  |  |  |
| Contamination factor | CF < 1 | Low contamination |
|  | 1 < CF ≤ 3 | Moderate contamination |
|  | 3 < CF ≤ 6 | Considerable contamination |
|  | 6 < CF | Very high contamination |
|  |  |  |
| Geoaccumulation index | I_geo_ ≤ 0 | Uncontaminated |
|  | 0 ≤ I_geo_ < 1 | Contaminated to moderately contaminated |
|  | 1 ≤ I_geo_ < 2 | Moderately contaminated |
|  | 2 ≤ I_geo_ < 3 | Moderately to strongly contaminated |
|  | 3 ≤ I_geo_ < 4 | Strongly contaminated |
|  | 4 ≤ I_geo_ < 5 | Strongly to extremely contaminated |
|  | 5 < I_geo_ | Extremely highly contaminated |
|  |  |  |
| Contamination degree | CD < 1.5 | Very low degree of contamination |
|  | 1.5 ≤ CD < 2 | Low degree of contamination |
|  | 2 ≤ CD < 4 | Moderate degree of contamination |
|  | 4 ≤ CD < 8 | High degree of contamination |
|  | 8 ≤ CD < 16 | Very high degree of contamination |
|  | 16 ≤ CD < 32 | Extremely high degree of contamination |
|  | 32 < CD | Ultra high degree of contamination |
|  |  |  |
| Metal load index | PLI < 1 | Unpolluted |
|  | PLI > 1 | Polluted |
|  |  |  |
| Potential ecological risk index | RI ≤ 150 | Low ecological risk |
|  | 150 < RI ≤ 300 | Moderate ecological risk |
|  | 300 < RI ≤ 600 | Considerable ecological risk |
|  | 600 < RI | Very high ecological risk |

**REFERENCES**

1. Muller, G. (1969). Index of geoaccumulation in sediments of the Rhine River. *GeoJournal*, *2* 108-118.

2. Weissmannová, H.D., & Pavlovský, J. (2017). Indices of soil contamination by heavy metals-methodology of calculation for pollution assessment (minireview). *Environmental Monitoring and Assessment*, *189*(12), 616. <https://doi.org/10.1007/s10661-017-6340-5>

3. Liao, J., Cui, X., Feng, H., & Yan, S. (2022). Environmental Background Values and Ecological Risk Assessment of Heavy Metals in Watershed Sediments: A Comparison of Assessment Methods. *Water*, *14*(1), 14010051. <https://doi.org/10.3390/w14010051>

4. Tomlinson, D.L., Wilson, J.G., Harris, C., & Jeffrey, D. (1980). Problems in the assessment of heavy-metal levels in estuaries and the formation of a pollution index. *Helgoländer Meeresuntersuchungen*, *33*566-575.
